# SARS-CoV-2 IgG spike protein antibody response in mRNA-1273 Moderna^®^ vaccinated patients on maintenance immunoapheresis – a cohort study

**DOI:** 10.3389/fimmu.2022.969193

**Published:** 2022-09-26

**Authors:** Martina Gaggl, Constantin Aschauer, Christof Aigner, Gregor Bond, Andreas Vychytil, Robert Strassl, Ludwig Wagner, Gere Sunder-Plassmann, Alice Schmidt

**Affiliations:** ^1^ Division of Nephrology and Dialysis, Department of Medicine III, Medical University of Vienna, Vienna, Austria; ^2^ Division of Clinical Virology, Department of Laboratory Medicine, Medical University of Vienna, Vienna, Austria

**Keywords:** covid-19, vaccination, IgG apheresis, immunoadsorption, SARS-CoV-2 IgG spike (S) protein antibody

## Abstract

**Background:**

The SARS-CoV-2 pandemic increased mortality and morbidity among immunocompromised populations. Vaccination is the most important preventive measure, however, its effectiveness among patients depending on maintenance immunoglobulin G (IgG) apheresis to control autoimmune disease activity is unknown. We aimed to examine the humoral immune response after mRNA-1273 Moderna^®^ vaccination in immunoapheresis patients.

**Methods:**

We prospectively monitored SARS-CoV-2 IgG spike (S) protein antibody levels before and after each IgG (*exposure*) or lipid (LDL) apheresis (*controls*) over 12 weeks and once after 24 weeks. Primary outcome was the difference of change of SARS-CoV-2 IgG S antibody levels from vaccination until week 12, secondary outcome was the difference of change of SARS-CoV-2 IgG S antibody levels by apheresis treatments across groups.

**Results:**

We included 6 IgG and 18 LDL apheresis patients. After 12 weeks the median SARS-CoV-2 IgG S antibody level was 115 (IQR: 0.74, 258) in the IgG and 1216 (IQR: 788, 2178) in the LDL group (p=0.03). Median SARS-CoV-2 IgG S antibody reduction by apheresis was 76.4 *vs.* 23.7% in the IgG and LDL group (p=0.04). The average *post- vs. pre*-treatment SARS-CoV-2 IgG S antibody rebound in the IgG group *vs.* the LDL group was 46.1 and 6.44%/week from prior until week 12 visit.

**Conclusions:**

IgG apheresis patients had lower SARS-CoV-2 IgG S antibody levels compared to LDL apheresis patients, but recovered appropriately between treatment sessions. We believe that IgG apheresis itself probably has less effect on maintaining the immune response compared to concomitant immunosuppressive drugs. Immunization is recommended independent of apheresis treatment.

## Introduction

In 2020 the new severe acute respiratory syndrome corona virus 2 (SARS-CoV-2) rapidly spread around the world and caused a pandemic with more than 500 million infections and 6 million deaths up until two years later (1). People with chronic disease and advanced age are at highest risk for severe course of the infection. The pandemic created a dilemma for patients with autoimmune disease on maintenance immunosuppression: On the one hand, immunosuppressive treatment makes them more susceptible to infection, and on the other hand any infection may trigger autoimmune disease episodes requiring enhancement of immunosuppression. Theoretically, among the most vulnerable are patients dependent on maintenance immunoglobulin G (IgG) apheresis in addition to immunosuppressive drug therapy. The extracorporeal treatment uses adsorption columns to selectively remove IgG antibodies and the depleted plasma is returned to the patient (2).

In early 2021 regulatory authorities approved several SARS-CoV-2 vaccinations targeting the spike protein expressed at the virus surface (3, 4). The efficacy of the messenger RNA vaccines and the conventional vaccines is excellent in the average healthy population and a universal recommendation for rapid vaccination has been issued. The Immunonephrology Working Group of the European Renal Association – European Dialysis and Transplant Association issued SARS-CoV-2 vaccination recommendations for patients receiving immunosuppressive therapies (5).

In several cohorts of immunocompromised patients impaired humoral immune response after complete SARS-CoV-2 immunization has been described (6–10).

We examined the humoral response to SARS-CoV-2 vaccination in our IgG apheresis cohort over twelve weeks and once at week 24. These patients receive a variety of immunosuppressants in addition to IgG apheresis and comprise a cohort of high-risk patients for severe SARS-CoV-2 infection. Our cohort of low-density lipoprotein (LDL) apheresis patients, who receive the identical extracorporeal procedure except for a different type of adsorption column selectively removing lipids from the plasma, serve as a control cohort.

Further we assessed the quantitative reduction of SARS-CoV-2 IgG spike (S) antibody levels by IgG apheresis compared to LDL apheresis sessions and the magnitude of recovery between treatment sessions. We hypothesize that by the nature of the treatment the maximum SARS-CoV-2 IgG S antibody levels will be lower and reduced by each IgG apheresis session, compared to subjects receiving LDL apheresis.

## Methods

### Study population

The study population consisted of patients enrolled in the chronic apheresis program of the Division of Nephrology and Dialysis, Department of Medicine III, of the Medical University at Vienna, Austria, as of February 2021. Patients either receive maintenance IgG or LDL apheresis. We included patients who were willed to participate in the study and gave informed consent. The study was approved by the ethics committee (No.: 3040/2021) of the Medical University of Vienna.

Subjects included in the study population were vaccinated on 11/MAR/2021 and 9/APR/2021, respectively, at the Medical University of Vienna. Two subjects of the IgG apheresis group received a 3^rd^ dose between week 12 and 24 visit.

We prospectively monitored SARS-CoV-2 IgG S antibody serum concentrations before and after each IgG apheresis (*exposure*) or LDL apheresis (*controls*) until week twelve after the first received dose of the vaccination. Laboratory assessment occurred as patients had regular treatment visits according to their treatment plan (**Supplemental Figure 1**). *Week 4 visit* was defined as the first treatment visit on or after 1^st^ of April prior to receiving the second dose of the vaccine, *week 8 visit* as the first treatment visit on or after 1^st^ of May but after having received the second dose of the vaccine, *week 12 visit* as the first treatment visit on or after 1^st^ of June, and *week 24 visit* as first treatment visit on or after 1^st^ of September 2021. Summaries of visits at exact time points (weeks one to twelve, after 11^th^ March 2021) are given in the supplemental material.

In case of more than one treatment per week we used the first occurring treatment to assess outcomes. To detect breakthrough SARS-Cov-2 infections subjects performed polymerase chain reaction (PCR) tests within 48 hours before each treatment visit and monthly SARS-CoV-2 nucleocapsid (N) protein tests.

### Outcomes

The *primary outcome* was the difference of change of SARS-CoV-2 IgG S antibody levels from the time of the first vaccination until twelve weeks across groups. A *secondary outcome* was the difference in reduction of SARS-CoV-2 IgG S antibody levels by apheresis treatment across groups. As *further outcomes* we evaluated SARS-CoV-2 IgG S antibody levels 24 weeks after the first immunization across groups. To differentiate between overall protein reduction and IgG antibody reduction by apheresis, we assessed the overall IgG antibody and albumin levels *pre-* and *post*-treatment at week four, eight, twelve and 24 of the study.

As a biomarker of immunocompetence we assessed peripheral blood copy numbers of the torque teno virus (TTV) at weeks eight, twelve, and 24.

### Apheresis

Plasma was separated from whole blood by centrifugation using the Spectra Optia^®^ (Terumo BCT, Lakewood, CO, USA) apheresis system (11). For anticoagulation citrate (ACD-A, anticoagulant citrate dextrose, formula A; Baxter, Munich, Germany) and sodium heparin (Heparin Immuno, Baxter-Immuno AG, Vienna, Austria; infusion rate: 1000 IE/h) was used. After separation from whole blood the plasma passed a Globaffin^®^ immunoadsorption column (Fresenius Medical Care Deutschland, Homburg, Germany) for IgG apheresis, which contains the peptide GAM 146 as a ligand directed against IgG antibodies (12). Lipoprotein apheresis was performed using the LDL-Therasorb^®^ (Therasorb, Munich, Germany) and LipoCollect^®^ column (Medicollect, Rimbach, Germany) to remove lipoprotein particles (12, 13).

Filtered plasma is then re-transfused together with the separated blood cells. Conservation and regeneration of the Globaffin^®^, Therasorb^®^, and LipoCollect^®^ column assigned explicitly to the same patient was performed according to standard procedures.

Five patients were treated by LDL apheresis using direct adsorption of lipoproteins from whole blood (DALI system, Fresenius Medical Care, St. Wendel, Germany) (14).

### Laboratory assessment

Antibodies against SARS-CoV-2 IgG S protein receptor binding domain (RBD) and the N antigen were measured in serum or plasma samples using a commercially available immunoassay (Elecsys^®^ Anti-SARS-CoV-2 IgG S, Roche Diagnostics, Mannheim, Germany). This assay uses a recombinant protein representing the RBD of the S antigen and the N antigen, respectively, in a double-antigen sandwich assay and was performed in the central laboratory of the Vienna General Hospital, Medical University of Vienna, according to the manufacturer’s instructions (15). The lower limit of detection (LLD) for SARS-CoV-2 IgG S antibodies was <0.04 U/mL, the upper limit of detection was 2500 U/mL, for SARS CoV-2 IgG N antibodies results were qualitative (positive/negative).

Total IgG levels were quantified by nephelometry (LLD: <195 mg/dL), serum albumin levels were measured by means of bromocresol green dye-binding methods, as detailed elsewhere (www.kimcl.at)

TTV DNA was extracted and quantified by means of PCR (7300 Real Time PCR System (Applied Biosystems, Foster City, CA), as described in detail elsewhere (16). Results were recorded in copies/mL.

### Data handling and statistical analysis

Percent change of antibody levels and albumin were calculated as “(pre-treatment level - post-treatment level) / pre-treatment level * 100)”. Antibody rebound per week between treatment session has been calculated as “(pre-treatment level - post-treatment level of last visit) / pre-treatment level)/ (days since last treatment/7) * 100)”.

Descriptive statistics were given as mean and standard deviation or median and interquartile range (IQR), as appropriate. Difference measures are presented with 95% confidence intervals (95%CI). Data distribution was analyzed using histograms. Individual and mean antibody concentrations were depicted as dot and line and smoothed line plots per week and study group. In case of missing data for respective time points, linear interpolation was used to estimate missing data.

Differences across groups were analyzed with the Mann-Whitney U test. Within subject changes have been compared with a Wilcoxon sign rank test. Correlations were assessed by the Spearman correlation coefficient.

## Results

### Study population

**Figure 1:**
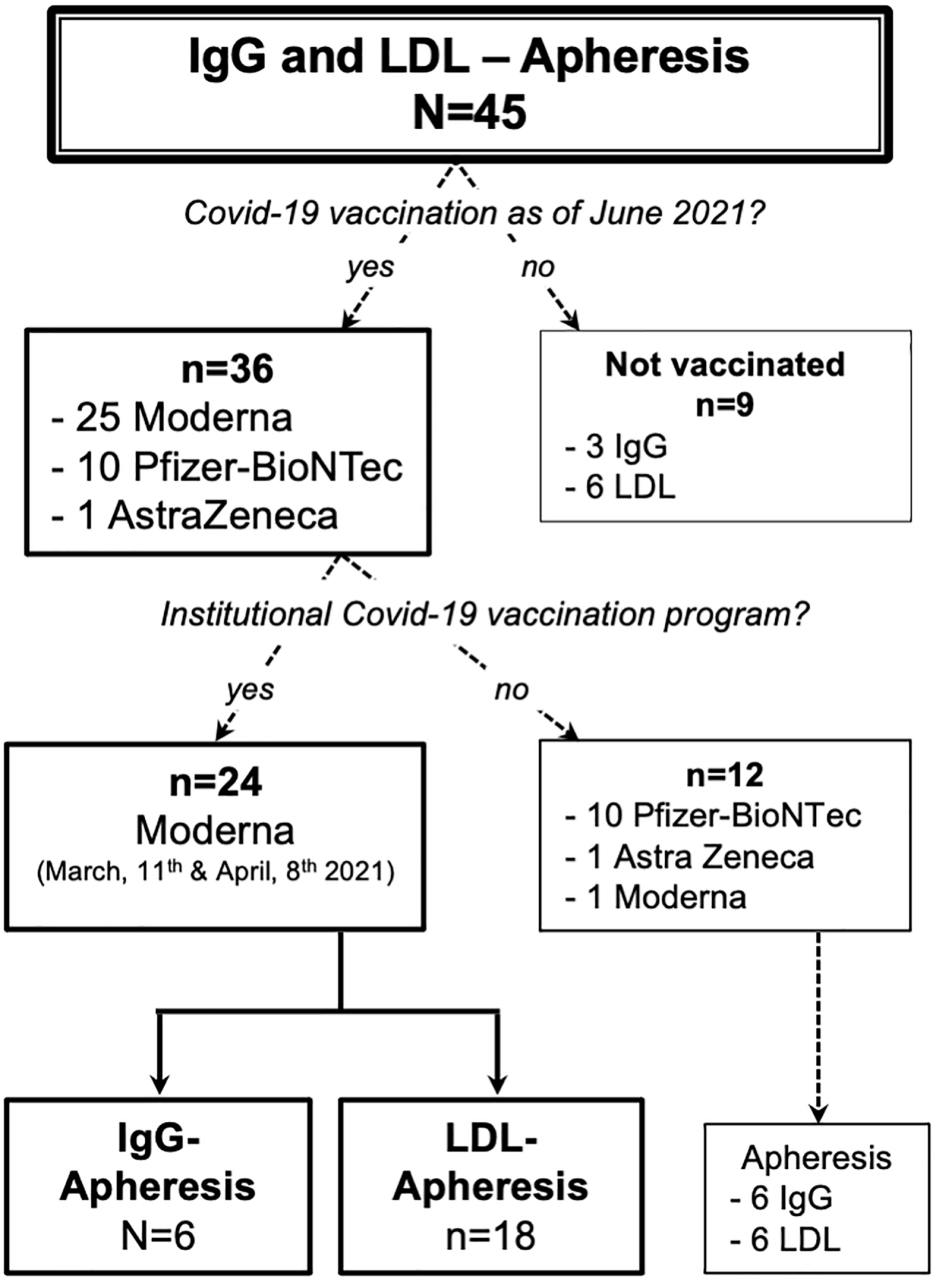
Composition of the study cohort.

As of March of 2021, 45 patients were enrolled in either the IgG or LDL apheresis program. As of June 2021, 36 patients had received one or two doses of a Covid-19 vaccine: 24 patients participated in our institutional vaccination program (**Figure 1**) and twelve received the vaccination elsewhere. Of the nine unvaccinated subjects, seven rejected the vaccination due to doubts with regard to safety and two wanted to use a different healthcare provider to receive the vaccine.

Basic demographic data of the apheresis cohort and the specific study cohort are given in **Tables 1**, **2**. The cohort consisted of six IgG apheresis and 18 LDL apheresis patients. IgG apheresis patients received one to four treatments per month, whereas the majority of LDL apheresis patients were treated weekly. Additional immunosuppressive medication is highlighted in **Table 2**. Except for one subject who had received a heart transplant in the past (azathioprine, cyclosporin, prednisolone; maintenance intravesical chemotherapy), none of the LDL apheresis patients received immunosuppressive medication. One LDL apheresis patient was human immunodeficiency virus (HIV) positive.

**Table 1:** Details of the Vienna apheresis cohort [count (percent), mean (standard deviation)] as of June 2021.

	IgG Apheresis	LDL Apheresis
N	15	30
Gender (female)	11 (73)	13 (43)
Age (years)	50 (11.3)	56 (16)
Vaccination (yes)	12 (80)	24 (80)
Hospital vaccination program	6 (40)	18 (60)
Other healthcare provider	6 (40)	6 (20)
Prior SARS-Cov-2 infection	2 (13)	3 (10)
Vaccinated	2 (13)	2 (7)

Hospital vaccination program: subjects were vaccinated on 11/MAR/2021 and 9/APR/2021, respectively, at the Medical University of Vienna; Other healthcare provider: subjects had received any SARS-Cov-2 vaccination elsewhere.

IgG, immunoglobulin G; LDL, low-density lipoprotein.

**Table 2:** Information on underlying main disease, treatment modalities, and disease specific treatments for the overall Viennese apheresis cohort (all) and the study cohort (study).

	Treatment	
	IgG Apheresis	
N	All (n=15)	Study (n=6)
Primary diagnosis		
*Myasthenia gravis*	9 (60)	4 (67)
*Systemic Lupus erythematosus*	3 (20)	1 (17)
*Chronic inflammatory demyelinating polyneuropathy*	2 (13)	1 (17)
*Pemphigus vulgaris*	1 (7)	0
Treatments per month	2 (1, 4)	1.25 (1, 1.88)
Treatment vintage (mL)	7200 (7200,7200)	7200 (7200,7200)
Type of adsorber		
*Globaffin* ^®^	14 (93)	6
*Therasorb* ^®^	1 (7)	0
Immunosuppressive medication		
*Prednisolone*	9 (60)	3 (50)
*Azathioprine*	7 (47)	3 (50)
*Mycophenolate mofetil*	1 (7)	1 (17)
*Rituximab*	3 (20)	1 (17)
*Eculizumab*	1 (7)	0
	**LDL Apheresis**	
N	All (n=30)	Study (n=18)
Primary diagnosis		
*Lipoprotein a excess*	22 (73)	15 (83)
*Hypercholesterolemia*	9 (30)	4 (22)
Concomitant disease		
*Peripheral vascular disease*	6 (20)	4 (22)
*Cardiovascular disease*	25 (83)	17 (94)
Treatments per month	4 (4, 4)	4 (4, 4)
Treatment vintage	6000 (6000, 6000)	6000 (6000, 6000)
Type of system/adsorber		
*Dali*	5 (17)	4 (22)
*LipoCollect300* ^®^	11 (37)	6 (33)
*Therasorb* ^®^	14 (47)	8 (44)
Specific medication		
*Evolocumab*	20 (67)	13 (72)
*Evinacumab*	2 (7)	0
*Alirocumab*	4 (13)	3 (17)

Numbers are given as median (interquartile range) and count (percent), respectively.

### SARS-CoV-2 IgG S antibody levels and change by treatment modality over 12 weeks

At week one all subjects of the study cohort tested negative for SARS-CoV-2 IgG nucleocapsid and SARS-CoV-2 IgG S antibodies. At week four visit the median *pre*-treatment SARS-CoV-2 IgG S antibody level was 3.49 (IQR: 0.66, 16.2) in IgG apheresis and 36.7 (IQR: 29.9, 164) U/mL in LDL apheresis patients (p=0.51, **Table 3**). IgG apheresis reduced the level by 64.6 (IQR: 34.5, 84.4) percent, whereas LDL apheresis resulted in a median reduction of 22.5 (IQR: 18.4, 28.9) percent (median difference (m_Δ_)= 36.38%; 95%CI: -19.74, 65.24; p=0.13). Of note, one LDL apheresis patient had received his second dose of the vaccine 6 days prior to his study visit week 4 (**Table 3**).

**Table 3:** SARS-CoV-2 IgG spike (S) protein antibody levels (U/mL), total IgG levels (mg/dL), and serum albumin levels (g/L) *pre vs. post* treatment and respective change (%) per treatment and time period after the first vaccination in 6 patients receiving IgG apheresis and 18 patients receiving LDL apheresis, respectively.

	Apheresis						
	IgG		LDL				
	*Pre*	*Post*	*Pre*		*Post*		*p-value*
***Week 4 visit***							
Median days	21 (20-22)		26 (26-27)				
SARS-CoV-2 IgG S	3.49 (0.66, 16.2)	0.72 (0.4, 2.22)	36.7^§^ (29.6, 164)		27.1 (18.8, 129)		
*Change (%)*	64.6 (34.5, 84.4)		22.5 (18.4, 28.9)				0.13
Total IgG	413 (363,522)	<195 (195, 195)	865 (700, 931)		635 (517, 782)		
*Change (%)*	52.1 (46.3, 61.4)		22.6 (16.9, 28.7)				<0.01
Serum albumin	41.6 (40.4, 43)	28.9 (28.4, 33.2)	40 (38.9, 40.7)		33.5 (31, 34.7)		
*Change (%)*	26.5 (22.2, 28.1)		18.1 (15.2, 21.5)				0.11
***Week 8 visit***							
Median days	54 (51-57)		56 (53-56)				
SARS-CoV-2 IgG S	192 (33.6, 847)	115 (6.05, 239)	1454 (859, 2403)		980 (250, 1839)		
*Change (%)*	40 (0, 80.2)		24.3 (16, 33.7)				0.92
Total IgG	382 (336, 646)	<195 (195, 195)	776 (690, 873)		606 (483, 750)		
*Change (%)*	49 (41.8, 67.5)		21.9 (18.2, 26.4)				0.02
Serum albumin	40.6 (40.2, 40.9)	34.5 (32.2, 38.5)	40.1 (39.8, 41.6)		32.1 (29.6, 34.3)		
*Change (%)*	15.6 (11.9, 20.7)		21 (16.4, 24.5)				0.12
***Week 12 visit***							
Median days	91 (85-94)		88 (83-91)				
SARS-CoV-2 IgG S	186 (29.3, 1074)	42.1 (6.1,165)	1216 (788, 2178)		839 (538, 1725)		
*Change (%)*	74.6 (52.7, 79)		23.7 (19.9, 27.3)				0.02
Total IgG	410 (335, 620)	<195 (195, 195)	861 (710, 998)		638 (535, 755)		
*Change (%)*	51.9 (41.6, 67.6)		25.8 (24, 29.5)				0.02
Serum albumin	41.2 (39.3, 42.2)	35.6 (33.9, 36.2)	40.9 (39.5, 42.4)		33.8 (30.8, 34.4)		
*Change (%)*	15.1 (13.2, 19.5)		19.4 (15.2, 21.4)				0.38
***Week 24 visit***							
Median days	184 (180, 190)		179 (176, 182)				
SARS-CoV-2 IgG S	498^*^ (78.2, 1126)	115 (14.2, 233)	656 (399,987)		468 (280, 735)		
*Change (%)*	76.5 (71.9, 79.1)		21.7 (16.2, 27)				0.13
Total IgG	408 (399, 746)	<195 (195, 195)	892 (739, 972)		654 (552, 767)		
*Change (%)*	52.2 (51.1, 73.9)		22.1 (19, 26.6)				<0.01
Serum albumin	43.1 (42.2, 44.9)	36.6 (35, 38.7)	40.6 (39.9, 42.4)		33.9 (32, 36.7)		
*Change (%)*	15.1 (10.2, 17.1)		15.7 (13.4, 20.9)				0.11

Median days, median days since the first vaccination at time of assessment; n, subjects assessed at respective time period; Numbers are given as median (interquartile range). P-values were calculated by using the Mann-Whitney-U test.

*Median pre-treatment SARS-CoV-2 IgG S antibody levels excluding 2 patients who had received 3 doses of the vaccine by study visit week 24: 498 (91.1, 974.5); ^§^ Median pre-treatment SARS-CoV-2 IgG S antibody levels excluding the subject who had received a second dose by study visit week 4: 35.6 (24.8, 152).

At week eight visit median *pre*-treatment SARS-CoV-2 IgG S antibody levels rose to 192 (IQR: 33.6, 847) in the IgG apheresis and to 1454 (IQR: 859, 2403) U/mL in the LDL apheresis group (m_Δ_= -939; 95%CI: -1964, -124; p=0.02). Change of SARS-CoV-2 IgG S antibody levels by apheresis treatment was 40 (IQR: 0, 80.2) and 24.3 (IQR: 16, 33.7) percent for IgG and LDL apheresis, respectively (m_Δ_= -3.36 x10^-5^; 95%CI: -24.76, 59.1; p=0.92).

At week twelve visit the median SARS-CoV-2 IgG S antibody level was 186 (IQR: 29.3, 1074) in the IgG and 1216 (IQR: 788, 2178; m_Δ_: -798; 95%CI: -1360, 21; p=0.09) U/mL in the LDL apheresis group. Median SARS-CoV-2 IgG S antibody reduction by apheresis treatment was 74.6 (IQR: 52.7, 79) and 23.7 (IQR: 19.9, 27.3) percent in the IgG and LDL apheresis group, respectively (m_Δ_=49.2; 95%CI: 20.43, 58.4; p=0.02).

After 12 weeks two females (33%) of the IgG group had SARS-CoV-2 IgG S antibody levels <0.75 U/mL compared to one woman (5.5%) from the LDL group.

**Figure 2:**
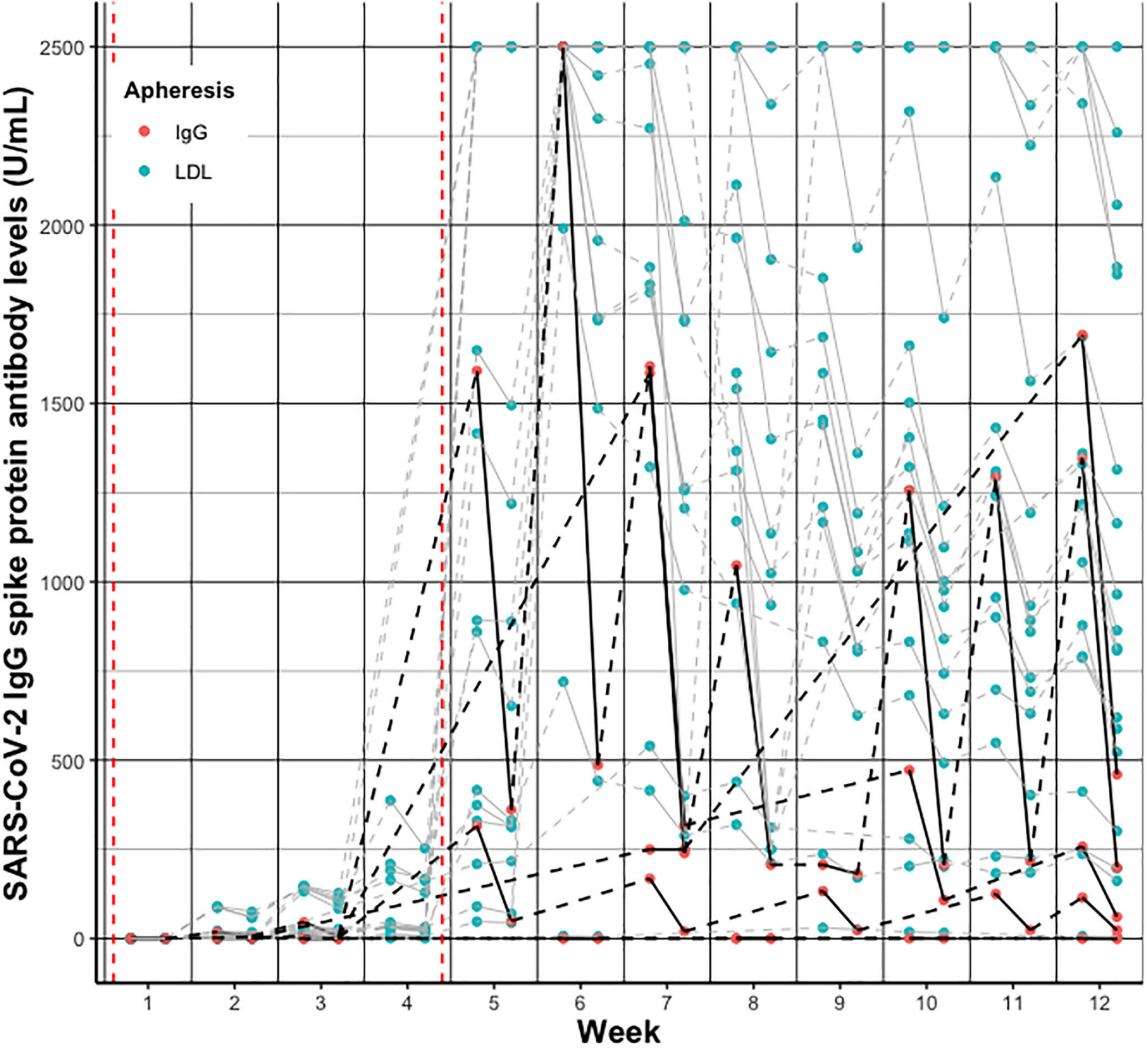
Individual change of SARS-CoV-2 IgG spike protein antibody levels *pre-* vs. post- vs. *pre-*treatment per week after vaccination in patients receiving IgG apheresis (black/red) and LDL apheresis (grey/blue). Solid lines indicate change from *pre*- to *post*-treatment levels, dashed lines indicate change from *post-* to next *pre-*treatment levels. Red dashed vertical lines indicate the first and second vaccination, respectively.

**Figure 3:**
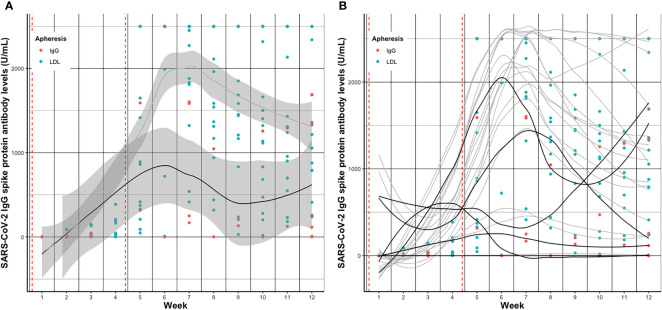
Smoothed curves (lines) of **(A)** average and **(B)** individual SARS-CoV-2 IgG spike protein antibody levels before apheresis treatment per week after vaccination in patients receiving IgG apheresis (black) and LDL apheresis (grey). Dots indicate individual exact SARS-CoV-2 IgG spike protein antibody levels (red, IgG; blue, LDL) per week. Red dashed vertical lines indicate the first and second vaccination, respectively. In **Figure 3B** geom_smooth function was used to draw smoothed curves. In case of missing measurements at time points values were extrapolated using the na.approx function of R. Dots indicate exclusively measured values.

Individual SARS-CoV-2 IgG S antibody levels *pre- vs. post- vs. pre*-treatment over the twelve weeks follow-up are depicted in **Figure 2** and separately displayed in **Supplemental Figures 2, 3**. Summarized SARS-CoV-2 IgG S antibody levels per week and group are depicted in **Figures 3A, B** and detailed in **Supplemental Table 1**.

### SARS-CoV-2 IgG S antibody levels, total IgG antibody levels, and serum albumin levels by treatment modality at visit week four, eight, twelve, and 24

**Figure 4:**
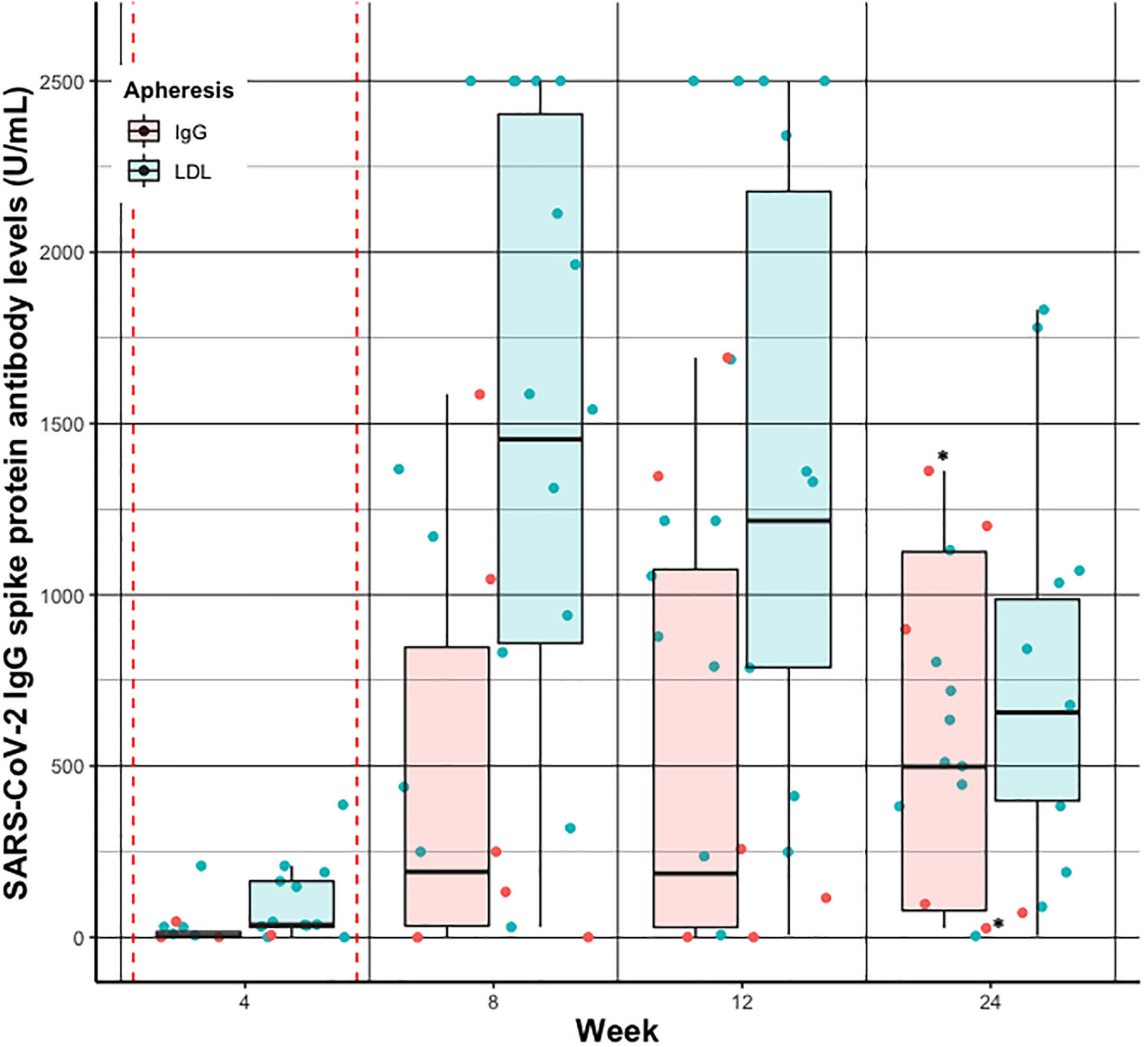
Individual (dots) and summarized (boxplots) SARS-CoV-2 IgG spike protein antibody levels before apheresis treatment at week 4, 8, 12, and 24 after vaccination in patients receiving IgG apheresis (red) and LDL apheresis (blue). Red dashed vertical lines indicate the first and second vaccination, respectively. * indicates subjects who have received a 3^rd^ dose of the vaccine. For better visualization the geom_jitter function has been used to present individual SARS-CoV- 2 IgG spike protein antibody levels.

SARS-CoV-2 IgG S antibody levels increased in both groups over time, and peaked around week five to seven, basically after the second vaccination (**Figure 3**). Median SARS-CoV-2 IgG S antibody levels were about 7.5 times higher in the LDL compared to the IgG apheresis group at week eight visit, and about 6.5 times higher at week twelve visit (**Table 3**). After 24 weeks, median SARS-CoV-2 IgG S antibody levels increased by about 2.7 times compared to week twelve, which was likely due to a third vaccination in two (33%) subjects that had antibody levels <0.75 U/mL after two doses. SARS-CoV-2 IgG S antibody levels were relatively stable in the remaining four IgG apheresis patients. On the contrary, median SARS-CoV-2 IgG S antibody levels in the LDL group decreased about 50% compared to the week twelve visit (**Figure 4**).

The median SARS-CoV-2 IgG S antibody level change *pre-* and *post-* treatment over the 24 weeks was on average 64% in the IgG and 23% in the LDL apheresis group. Change of total IgG antibody levels and serum albumin levels by LDL apheresis were approximately similar compared to the change of SARS-CoV-2 IgG S antibody levels. As expected, the reduction of total IgG per treatment session was significantly higher in the IgG apheresis group (**Table 3**), and comparable to SARS-CoV-2 IgG S antibody level change per treatment session. Notably, all patients of the IgG apheresis group reached the LLD of total IgG levels after each IgG apheresis (<195 mg/dL). Since the exact level of total IgG antibody concentrations were not available the true percent reduction couldn’t be calculated. Subsequently, change of total IgG concentrations were less than the change of SARS-CoV-2 IgG S antibody levels per treatment (approximately 50 vs. 65%).

### Rebound of SARS-CoV-2 IgG S antibody levels between treatment sessions

The average *post-* vs. *pre*-treatment SARS-CoV-2 IgG S antibody rebound was 119 (IQR: 75, 139) and 136 (IQR: 126, 139) percent per week in the IgG compared to the LDL apheresis group from week four until next visit (**Supplemental Figures 3A vs. B**). As expected, this steep increase flattened to 50.3 (IQR: 27.1, 63.9) *vs.* -2.77 (IQR: -28.1, 39.8) percent per week from week eight until next visit, and 46.1 (IQR: 40.3, 48.6) *vs.* 6.44 (IQR: 3.83, 11.8) percent per week from prior visit until week twelve visit in the IgG *vs.* the LDL group.

The spread of individual antibody concentrations is relatively large across the six individuals receiving IgG apheresis (**Supplemental Figure 2A**). Two subjects did not develop SARS-CoV-2 IgG S antibody levels until week twelve visit (both steroid, 1 mycophenolate mofetil, 1 azathioprine). Two subjects had levels < 500 U/mL; one on maintenance azathioprine therapy. Only 1 reached the upper limit of antibody quantification (>2500 U/mL) at week six, and had levels above 1000 U/mL at week twelve. Notably, this lady is on azathioprine and steroid treatment, had weekly apheresis treatments at that time and received her last dose of rituximab in December 2020. One patient had antibodies above 1500 U/mL at *pre*-treatment determination. However, independent of the absolute magnitude of antibodies and elapsed time between treatment sessions, individuals recovered to their “personal” SARS-CoV-2 IgG S antibody level until the next treatment session.

In contrast, the majority (78%) of LDL apheresis patients reached the >2500 U/L SARS-CoV-2 IgG S antibody level at week six, and eleven were above 1000 U/L at week twelve. As expected, subjects recovered to comparable SARS-CoV-2 IgG S antibody levels between treatment sessions. Individual levels are shown in **Figure 2** and **Supplemental Figures 2B, 3B**. Notably, only one subject in the LDL group did not develop SARS-CoV-2 IgG S antibodies (azathioprine, cyclosporin, prednisolone; maintenance intravesical chemotherapy), three subjects had levels < 500 U/mL (1 severe liver disease, 1 diabetes, 1 arterial hypertension) until week twelve.

Interestingly, in the IgG apheresis group median SARS-CoV-2 IgG S antibody levels remained around a similar concentration from week twelve visit until week 24 visit (p=0.69), but decreased in the LDL apheresis group about 500 U/mL from week twelve visit until week 24 visit (p<0.01). Of note, two subjects from the IgG apheresis group received a third dose of vaccination between week twelve and 24.

### SARS-CoV-2 IgG S antibody levels in IgG apheresis patients not included in the study cohort

Of the 15 IgG apheresis patient treated at our facility, three were vaccinated prior to the hospital-initiated vaccination program, and three later. Two had a SARS-CoV-2 infection right before and after the vaccination, respectively, and all received two doses of the Pfizer-BioNTech vaccine. Twelve weeks after the vaccination the median *pre*-treatment SARS-CoV-2 IgG S antibody levels were 1324 (IQR: 307, 2215) U/mL. All had detectable antibody levels, two had levels < 500 U/mL (1 rituximab 02/2020), three had levels >2000 U/mL prior to the apheresis treatment (1 rituximab + azathioprine + steroids, 1 azathioprine + eculizumab). Taken together, in all twelve IgG apheresis patients the median *pre*-treatment SARS-CoV-2 IgG S antibody level was 471 (IQR: 153, 1760) U/mL twelve weeks after the immunization.

### SARS-CoV-2 IgG S antibody levels in relation to TTV copy numbers

The highly prevalent and non-pathogenic TTV is an emerging marker to monitor immunosuppression (17, 18). Median average TTV loads from week eight until 24 in the IgG apheresis group were 4.0 x 10^4^ (IQR: 5.6 x 10^3^, 9.1 x 10^4^) and 5.2 x 10^4^ (IQR: 2.9 x 10^4^, 2.6 x 10^5^) copy numbers in the LDL apheresis group. There was no association between individual TTV copy numbers and *pre*-treatment SARS-CoV-2 IgG S antibody levels at week eight, twelve, and 24 in both groups.

## Discussion

Our study is the first prospective monitoring of SARS-CoV-2 IgG S antibody levels after an immunization with the mRNA-1273 Moderna^®^ vaccine in subjects receiving maintenance apheresis treatment. Although this concerns a relatively small population, information on the effect of IgG apheresis on immune response after vaccinations in such populations in general is scarce. Concerns whether the apheresis treatment interferes with the vaccine response are present among patients and physicians. Indisputable is the fact that patients who are dependent on IgG apheresis comprise one of the most vulnerable populations with regard to infections. Preventive interventions such as vaccinations should be of highest priority in such cohorts.

First, we found that twelve weeks after full immunization subjects receiving LDL apheresis had on average higher levels of SARS-CoV-2 IgG S antibody levels compared to the IgG apheresis group. The initial antibody peak after the second dose of the vaccine was higher in the LDL compared to the IgG apheresis group. After 6 months SARS-CoV-2 IgG S antibody levels approximately remained at the level of week twelve in the IgG apheresis group, but decreased about 50% in the LDL apheresis group. This observation might be due to a third vaccination in two primary non-responders in the IgG apheresis group. However, at least two IgG subjects (50%) had unchanged concentrations after 24 weeks.

Second, we demonstrated that IgG apheresis decreased the SARS-CoV-2 IgG S antibody levels by about 64% per treatment session. This is comparable with the total IgG reduction per treatment and according to results reported by others (19). However, due to the lower detection limits of our laboratory we could not exactly calculate total IgG decrease. In contrast, changes of antibody levels in LDL apheresis patients can be interpreted as an overall protein decrease due to plasma dilution and protein wasting caused by the extracorporeal procedure.

Third, SARS-CoV-2 IgG S antibody levels rebounded in each subject to levels measured prior to the treatment until the next treatment visit. Treatment frequency had no obvious effect on the antibody rebound. Noteworthy, timing of specimen determination plays a crucial role when calculating the antibody rebound. All subjects had at least one week between the last treatment session and determination of the subsequent *pre*-treatment antibody level. Interestingly, after full immunization subjects seemed to develop a “personal” SARS-CoV-2 IgG S antibody level, which remained stable independent of treatment frequency over the twelve weeks of follow-up. Thus, we believe that apheresis itself has little effect on the development of SARS-CoV-2 IgG S antibodies and whether *pre*-treatment levels are in the higher or lower range of the measurable spectrum.

Our study cohort comprises a mixed repertoire of immunocompromised patients, either due to comorbidities, such as liver or metabolic disease, or due to additional immunosuppressive medications. TTV, a highly prevalent and non-pathogenic small nonenveloped DNA virus, is an emerging marker to monitor immunosuppression in recipients of solid organ transplantations (17, 20) and in patients with rheumatologic disease (21). Moreover, TTV is associated with immunocompetence in patients with HIV (22) and with response to SARS-CoV-2 vaccination in immunocompromised patients (18, 23). TTV copy numbers in our sample, independent of type of apheresis, were comparable to those in healthy population, suggesting a less immunocompromised cohort compared to mentioned reference cohorts. Subsequently, we saw no association between TTV copy numbers and the SARS-CoV-2 IgG S antibody response in our cohort. However, it’s also worth mentioning that the effect of immunoapheresis on TTV copy numbers is yet unknown.

It is well documented that patients receiving immunosuppressive medications, especially antimetabolites (mycophenolate mofetil/mycophenolic acid/azathioprine), have a drastically reduced seroconversion rate compared to patients not receiving such medications (24–28). Depending on the number of applied injections and the type of vaccine used the seroconversion rate after 28 days varied between 5.7 and 62% (29). Furthermore, B-cell depleting anti-CD20 therapies jeopardizes the effectiveness of T- and B-cell conversion after vaccinations. In a meta-analysis overall only 40% of patients adequately responded after vaccination (30). However, time since last anti-CD20 therapy and indication significantly modified associations: Only 20% of patients, who received a treatment within last six months responded, compared to 63% who did not receive a treatment within the last six months. B-cell depletion was a strong predictor for non-responders (20% vs. 77%). Overall cell-mediated immune response was 73% (95%CI: 57%, 87%), but varied across studies (44% to 100%) (30).

Conclusions drawn from our study are limited by the rather small sample size of IgG apheresis patients. Furthermore, the population is highly heterogenous with regard to underlying disease and immunosuppression. Since the introduction of more targeted treatments, multiple subjects could be successfully weaned off the extracorporeal treatment, and only a small sample depends on the continuation of this rather unspecific chronic treatment option. Subsequently, studies with larger sample sizes are not feasible in a single center study design. However, by including our six patients that received a SARS-CoV-2 vaccination through a different healthcare provider at an earlier or later time point than our study cohort, we demonstrated that antibody response, at least twelve weeks after the vaccination, is approximately comparable to those of the prospectively followed cohort. Interestingly, median antibody response in those 6 patients was more distinct, which might be due to concomitant native SARS-CoV-2 infections and/or related to the different type of vaccination applied.

One subject of the LDL apheresis group violated the protocol by receiving his second dose of the vaccine 6 days prior to his study visit week 4. Although his individual antibody levels significantly rose compared to his last measurement prior to the second dose (0.4 to 209 U/mL), the overall results and interpretation of the data didn’t change when the individual was excluded.

In conclusion, IgG apheresis patients had lower SARS-CoV-2 IgG S antibody levels compared to LDL apheresis patients, but SARS-CoV-2 IgG S antibody levels appropriately rose to *pre-*treatment levels between treatment sessions independent of treatment frequency. Thus, we believe that IgG apheresis itself has little effect on an adequate maintenance of an immune response after Covid-19 vaccination and strongly recommend preventive vaccination independent on apheresis treatment schedules.

## Data availability statement

The data underlying this article will be shared on reasonable request to the corresponding author.

## Ethics statement

The studies involving human participants were reviewed and approved by Ethikkommission der Medizinischen Universität Wien. The patients/participants provided their written informed consent to participate in this study.

## Author contributions

MG, AS, GB and GS-P designed the study, MG, AS, CAs, CAi conducted the study, RS and LW performed laboratory analysis; MG and AS analyzed data and drafted the manuscript, GB, GS-P, AS, CAs, CAi, RS, AV, GB, and LW critically proof-read the manuscript. All authors contributed to the article and approved the submitted version.

## Acknowledgments

We acknowledge the help of all nurse staff of the apheresis unit of the Department of Medicine III, Division of Nephrology and Dialysis, at the Medical University of Vienna in managing patients and collecting blood samples.

## Conflict of interest

The authors declare that the research was conducted in the absence of any commercial or financial relationships that could be construed as a potential conflict of interest.

## Publisher’s note

All claims expressed in this article are solely those of the authors and do not necessarily represent those of their affiliated organizations, or those of the publisher, the editors and the reviewers. Any product that may be evaluated in this article, or claim that may be made by its manufacturer, is not guaranteed or endorsed by the publisher.
