# Supplementary material for: SARS-CoV-2 IgG spike protein antibody response in mRNA-1273 Moderna® vaccinated patients on maintenance immunoapheresis – a cohort study
Source: Front Immunol. 2022 Sep 26;13:969193. doi: 10.3389/fimmu.2022.969193 (PMC9549982; doi:10.3389/fimmu.2022.969193)
Supplement: Supplementary file 1 [file DataSheet_1.docx]

**Supplemental Material: SARS-CoV-2 IgG Spike Protein Antibody Response in mRNA-1273 Moderna^®^** **vaccinated Patients on maintenance Immunoapheresis – a Cohort Study**

By Gaggl *et al*

**Supplemental table 1.** Total SARS-CoV-2 IgG spike (S) protein levels (U/mL) *pre vs. post* treatment and respective percent (%) change per week after the first vaccination in patients receiving IgG apheresis and LDL apheresis, respectively.

|  |  | **Apheresis** | | | | |
| --- | --- | --- | --- | --- | --- | --- |
|  |  | **IgG** | | **LDL** | |  |
|  | | *Pre* | *Post* | *Pre* | *Post* | *p-value* |
| ***Week 1*** |  | n=1 |  | n=14 |  |  |
| SARS-CoV-2 IgG S | | 0.4 (0.4,0.4) | 0.4 (0.4, 0.4) | 0.4 (0.4, 0.4) | 0.4 (0.4, 0.4) |  |
| *Change (%)* | | 0 | | 0 | | n.a. |
| ***Week 2*** |  | n=4 |  | n=18 |  |  |
| SARS-CoV-2 IgG S | | 0.97 (0.4,5.77) | 0.9 (0.4, 2.88) | 1.98 (0.4, 8.92) | 1.56 (0.4, 6.44) |  |
| *Change (%)* | | 4.58 (0, 21.9) | | 15.8 (0, 26.4) | | 0.63 |
| ***Week 3*** |  | n=2 |  | n=14 |  |  |
| SARS-CoV-2 IgG S | | 0.57(0.49, 0.66) | 0.4 (0.4, 0.4) | 28.4 (14.6, 137) | 20.2 (10.1, 95.9) |  |
| *Change (%)* | | 23 (11.5, 34.5) | | 29.2 (21.8, 35.5) | | 1.00 |
| ***Week 4*** |  | n=2 |  | n=11 |  |  |
| SARS-CoV-2 IgG S | | 26.2 (16.2, 36.2) | 3.4 (2.22, 4.58) | 36.7 (29.9, 177) | 30.1 (20.4, 146) |  |
| *Change (%)* | | 85.4 (84.4, 86.5) | | 27.5 (19.9, 33.1) | | 0.03 |
| ***Week 5*** |  | n=1 |  | n=19 |  |  |
| SARS-CoV-2 IgG S | | 1591 | 361 | 374 (41, 1532) | 314 (34.9, 1357) |  |
| *Change (%)* | | 77.3 | | 13.9 (0.17, 23.5) | | 0.12 |
| ***Week 6*** |  | n=4 |  | n=18 |  |  |
| SARS-CoV-2 IgG S | | 158(0.04, 862) | 24.8 (0.04, 159) | 2500 (2500, 2500) | 2500 (1794, 2500) |  |
| *Change (%)* | | 40.3 (0, 81.5) | | 0 (0, 22.5) | | 0.38 |
| ***Week 7*** |  | n=3 |  | n=16 |  |  |
| SARS-CoV-2 IgG S | | 1585 (876, 1594) | 240 (130, 278) | 2475 (1828, 2500) | 1874 (1244, 2500) |  |
| *Change (%)* | | 85 (82.5, 86.6) | | 21.5 (0, 29.6) | | 0.01 |
| ***Week 8*** |  | n=3 |  | n=16 |  |  |
| SARS-CoV-2 IgG S | | 250 (125, 648) | 207 (104, 228) | 1775 (1276, 2500) | 1080 (250, 2053) |  |
| *Change (%)* | | 0 (0, 40.1) | | 25.1 (12.2, 43.7) | | 0.46 |
| ***Week 9*** |  | n=2 |  | n=13 |  |  |
| SARS-CoV-2 IgG S | | 104 (52, 155) | 90.7 (45.6, 136) | 1686 (1167, 2500) | 1192 (805, 2500) |  |
| *Change (%)* | | 6.28 (3.14, 9.42) | | 24.8 (0, 29.3) | | 0.34 |
| ***Week 10*** |  | n=3 |  | n=21 |  |  |
| SARS-CoV-2 IgG S | | 133(67, 694) | 23 (11.7, 114) | 1441 (1114, 2500) | 1030 (743,2500) |  |
| *Change (%)* | | 82.7 (41.4, 83.2) | | 22.5 (0, 27.9) | | 0.22 |
| ***Week 11*** |  | n=3 |  | n=17 |  |  |
| SARS-CoV-2 IgG S | | 472 (298, 882) | 108 (65.8, 162) | 1310 (901, 2500) | 934 (692, 2224) |  |
| *Change (%)* | | 81 (79.1, 82.1) | | 16.7 (2.6, 26.8) | | <0.01 |
| ***Week 12*** |  | n=6 |  | n=18 |  |  |
| SARS-CoV-2 IgG S | | 186 (29.3, 1074) | 42.1 (6.1, 164) | 1216 (788, 2178) | 839 (538, 1725) |  |
| *Change (%)* | | 74.6 (52.7, 79) | | 23.7 (19.9, 27.3) | | 0.02 |

**Supplemental figure legends**

**Supplemental Figure 1.** Time plan of the study.

**Supplemental Figure 2.** Individual *pre- vs. post*-treatment changes of SARS-CoV-2 IgG spike protein antibody levels over time in patients receiving (A) IgG apheresis and (B) LDL apheresis. Red dashed vertical lines indicate the first and second vaccination, respectively.

**Supplemental Figure 3.** Individual *post- vs. f*ollowing *pre*-treatment changes of SARS-CoV-2 IgG spike protein antibody levels over time in patients receiving (A) IgG apheresis and (B) LDL apheresis. Red dashed vertical lines indicate the first and second vaccination, respectively.

**Supplemental figure 1.**

**
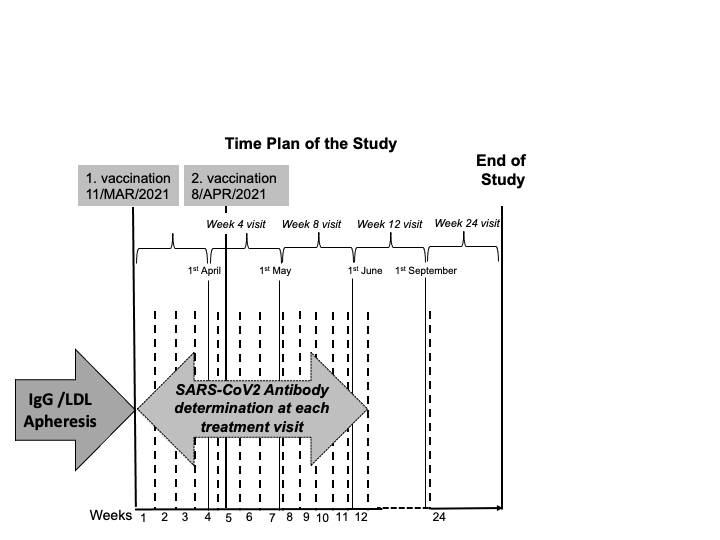
**

**Supplemental figure 2.**

| **(A)** | **(B)** |
| --- | --- |
| **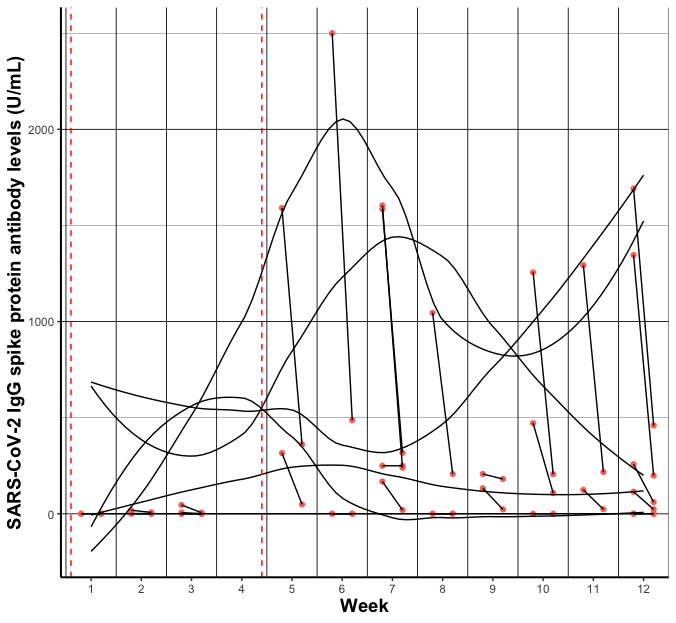** | **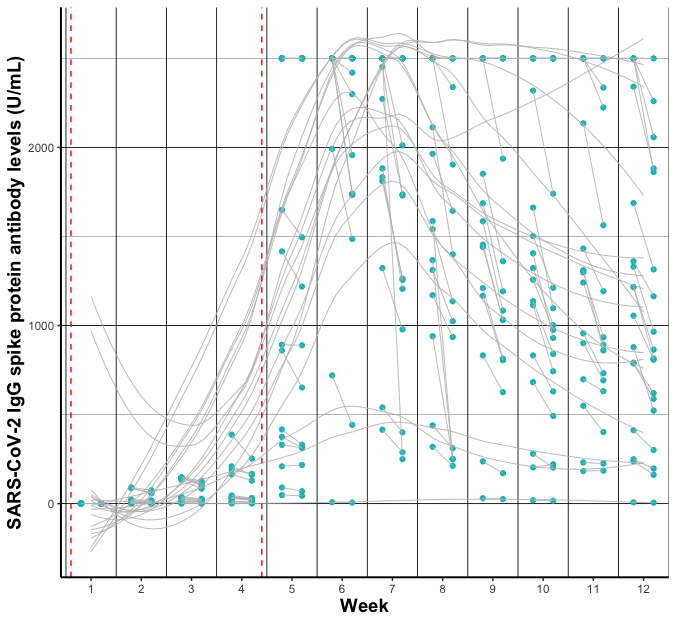** |
| Dots indicate exact measurements, dashed smoothed curves depict estimated follow-up over time (*geom_smooth* function of R). For smoothed curves missing measurements were extrapolated using the *na.approx* function of R. | |

**Supplemental figure 3.**

| **(A)** | **(B)** |
| --- | --- |
| **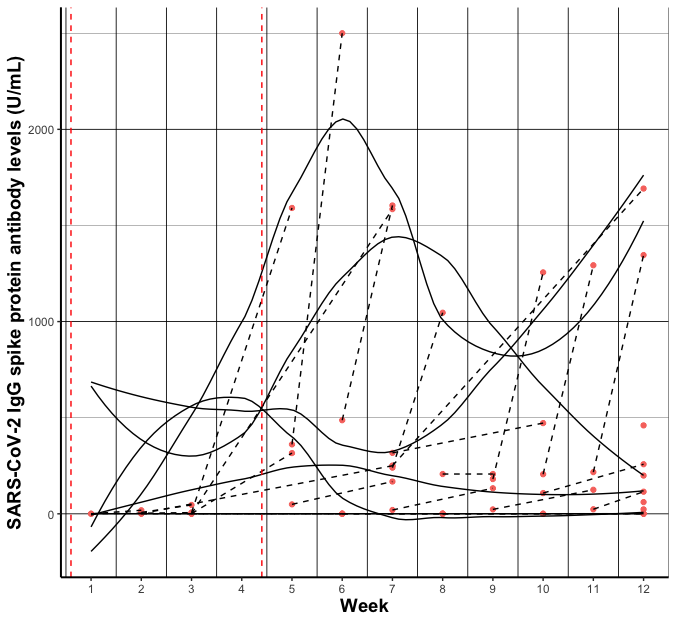** | **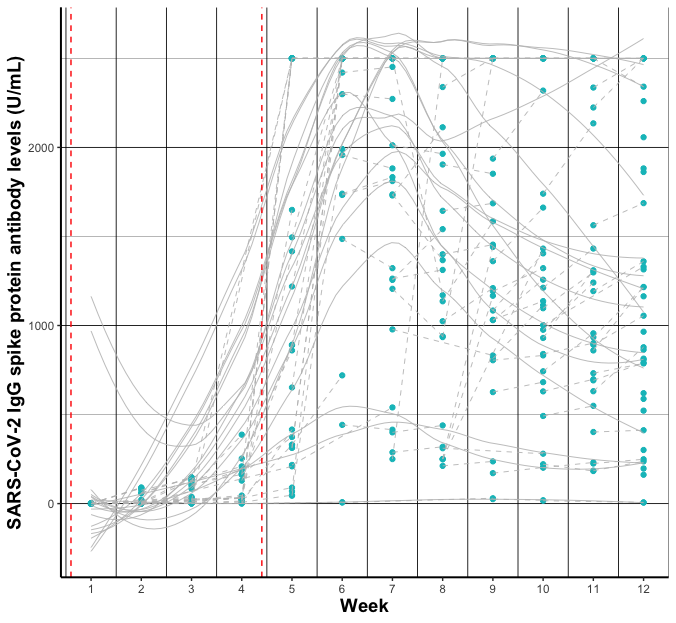** |
| Dots indicate exact measurements, dashed smoothed curves depict estimated follow-up over time (*geom_smooth* function of R). For smoothed curves missing measurements were extrapolated using the *na.approx* function of R. | |
